# Africanized Bee Venom (*Apis mellifera* Linnaeus): Neuroprotective Effects in a Parkinson’s Disease Mouse Model Induced by 6-hydroxydopamine

**DOI:** 10.3390/toxics10100583

**Published:** 2022-10-03

**Authors:** Camila G. Dantas, Ailma O. da Paixão, Tássia L. G. M. Nunes, Italo J. F. Silva, Bruno dos S. Lima, Adriano A. S. Araújo, Ricardo L. C. de Albuquerque-Junior, Kátia P. Gramacho, Francine F. Padilha, Luiz P. da Costa, Patricia Severino, Juliana C. Cardoso, Eliana B. Souto, Margarete Z. Gomes

**Affiliations:** 1 Institute of Research and Technology, Tiradentes University, Av. Murilo Dantas, 300, Aracaju 49032-490, Sergipe, Brazil; 2 Department of Pharmacy, Federal University of Sergipe (U.F.S.), Cidade Universitária Prof. José Aloísio de Campos, Av. Marechal Rondon, Jardim Rosa Elze, São Cristóvão 49100-000, Sergipe, Brazil; 3 Department of Pathology, Federal University of Santa Catarina, Florianópolis 88040-370, Santa Catarina, Brazil; 4 Department of Animal Science, Rural Federal University of Semi-Árido (U.F.E.R.S.A), Av. Francisco Mota, Costa e Silva, Mossoró 49032-490, Natal, Brazil; 5 Post-Graduation Program in Chemistry, Federal University of Sergipe (U.F.S.), Cidade Universitária Prof. José Aloísio de Campos, Av. Marechal Rondon, Jardim Rosa Elze, São Cristóvão 49100-000, Sergipe, Brazil; 6 Department of Pharmaceutical Technology, Faculty of Pharmacy, University of Porto, Rua de Jorge Viterbo Ferreira, 228, 4050-313 Porto, Portugal; 7 REQUIMTE/UCIBIO, Faculty of Pharmacy, University of Porto, Rua de Jorge Viterbo Ferreira, 228, 4050-313 Porto, Portugal

**Keywords:** 6-hydroxydopamine, bee venom, Parkinson’s disease, mice model, neuroprotection

## Abstract

This study evaluated the neuroprotective effects of the Africanized bee venom (BV) and its mechanisms of action after 6-hydroxydopamine-(6-OHDA)-induced lesion in a mice model. Prior to BV treatment, mice received intrastriatal microinjections of 6-OHDA (no induced dopaminergic neuronal death) or ascorbate saline (as a control). BV was administered subcutaneously at different dosages (0.01, 0.05 or 0.1 mg·Kg^−1^) once every two days over a period of 3 weeks. The open field test was carried out, together with the immunohistochemical and histopathological analysis. The chemical composition of BV was also assessed, identifying the highest concentrations of apamin, phospholipase A_2_ and melittin. In the behavioral evaluation, the BV (0.1 mg·Kg^−1^) counteracted the 6-OHDA-induced decrease in crossings and rearing. 6-OHDA caused loss of dopaminergic cell bodies in the substantia nigra pars compacta and fibers in striatum (STR). Mice that received 0.01 mg·Kg^−1^ showed significant increase in the mean survival of dopaminergic cell bodies. Increased astrocytic infiltration occurred in the STR of 6-OHDA injected mice, differently from those of the groups treated with BV. The results suggested that Africanized BV has neuroprotective activity in an animal model of Parkinson’s disease.

## 1. Introduction

The nigrostriatal pathway is an important dopaminergic pathway, and its imbalance can lead to different disease states such as Parkinson’s disease (PD), where dopamine depletion results in motor, cognitive and/or emotional impairments [1]. A rodent model of cell death that induces gradual loss of dopaminergic neurons in the substantia nigra pars compacta (SNc) and dopamine depletion in the striatum (CPu) is the intrastriatal microinjection of 6-hydroxydopamine (6-OHDA). It has been widely used for the evaluation of potential neuroprotective agents [2].

The pharmacological treatments available for PD aim to restore dopaminergic activity to improve functional mobility, thus increasing patient’s quality of life [3]. The main drug used as an attempt to replace the lack of dopamine is 3,4-dihydroxyphenylalanine (Levodopa or L-DOPA); however, the benefits of such pharmacotherapy are reduced over time and promote debilitating side effects called dyskinesias [4,5].

Previous studies reported neuroprotective actins of European bee venom (BV) by using acute models of PD [6,7,8]. However, in progressive dopaminergic lesions, such as that induced by intrastriatal 6-OHDA that effectively mimics the disease evolution, the implications on glial cells, especially astrocytes, which are fundamental in neuroinflammatory conditions, have not been elucidated yet.

In addition, the Africanized BV (*Apis mellífera* Linnaeus) presents different chemical composition when compared to European BV [9,10], and its active compounds have been used against a variety of neurodegenerative diseases, mainly because of their action on neuroinflammatory responses [11].

BV consists of a mixture of enzymes, peptides and amino acids, among others, with potent activity on neurological disorders [7,11,12]. The protein melittin, equivalent to 50% of the BV’s dry weight, has been linked to dopaminergic transmission, and along with apamin, another BV peptide compound that acts as a Ca^+^-activated K^+^ channel blocker, may cause increased activity of dopaminergic neurons [6]. 

With this work, we aimed to evaluate the neuroprotective effects of Africanized BV (*Apis mellífera* L.) and related mechanisms of action after a 6-OHDA-induced chronic and progressive dopaminergic injury in a rodent model of Parkinson’s disease (PD).

## 2. Materials and Methods

### 2.1. Chemical Analysis by High-Performance Liquid Chromatography Combined with Diode Array (HPLC-DAD)

The BV (10 mg) was diluted with 10 mL trifluoroacetic acid (0.1% in milli-Q water). The solution (concentration: 1 mg·mL^−1^) was centrifuged at 12,000 rpm for 10 min (4 ºC), and all the supernatant (approximately 9 mL) was collected for analysis by HPLC. Before high-performance liquid chromatography (HPLC) injections, the sample was filtered in membrane filter (0.45 µm PTFE, Sigma–Aldrich, St. Louis, MO, USA). The HPLC analysis was performed using a high-performance liquid chromatography system that consisted of a degasser DGU-20A3, two LC-20AD pumps, an SIL-20A HT auto injector, CTO-20A column oven, SPDM20Avp photodiode array detector (DAD) and a CBM-20A system controller (Shimadzu Co., Kyoto, Japan). The chromatographic separation was performed using analytical column C18 Phenomenex Luna® 4.6 × 250 mm (5 µm particle size). 

The mobile phase consisted of: A: trifluoroacetic acid (0.1% in water milli-Q) and B: acetonitrile. The mobile phase flow rate was 0.8 mL·min^−1^, and the sample injection volume was 20 µL. The elution gradient started with 5% B for 7 min, 5–10% B during 7–10 min, 10–17% B during 10–15 min, 17–25% B during 15–20 min, 25–35% B during 20–33 min, 35–40% B during 33–39 min and 40–5% B during 39–45 min, returning to the initial conditions and finalizing the analysis. The detector (HPLC—DAD, Shimadzu Co., Kyoto, Japan) was set at 280 nm for the peaks analysis. The compounds were identified through standard co-injections comparing the retention times. 

Apamin, melittin and phospholipase A_2_ were obtained from Sigma-Aldrich^®^ (St. Louis, MO, USA) and diluted with water milli-Q until 200 µg·mL^−1^ (stock solution). The samples were filtered in membrane filter (0.45 µm—PTFE), before HPLC injections. The quantitative analysis was performed through calibration curves of each compound standard identified. The curves were prepared in seven different concentrations: 0.8, 4, 8, 12, 16, 20 and 50 µg·mL^−1^. All samples were filtered in membrane filter (0.45 µm—PTFE) before HPLC injection and were analyzed in triplicate [12].

### 2.2. Apis mellífera Linnaeus Bee Venom and Other Chemicals

The bee venom in powder form was purchased from an apiary in the city of São Roque (São Paulo, Brazil), in the Southeast region of Brazil (23° 31′ 51″ S and 47° 8′ 8″ W). It was obtained by electrical extraction. The technique consists of placing a collector, composed of glass plates and pulse generator, connected to a source of energy (battery), in the hive entry. When the bees land on the plate, they receive a shock and react in order to sting the electrical collector plate, depositing a charge of poison between the glass and the protective material of the equipment. There it dries and then is scraped. Once acquired, the venom (powder) was diluted in sterile saline solution (0.9%, vehicle), obtaining a pH 5.0. After dilution, the BV was stored in an amber bottle and under refrigeration (10 °C). Other commercial chemicals used were: 6-hidroxidopamin (Sigma-Aldrich^®^, São Paulo, Brazil), Bovine serum albumin (BSA, Sigma-Aldrich^®^, São Paulo, Brazil), avidin-biotin peroxidase system (Kit ABC, Vectastain, Vector Laboratories®, São Paulo, Brazil), Diaminobenzidine (DAB) (Sigma-Aldrich^®^, São Paulo, Brazil). The primary antibodies used were rabbit anti-tyrosine hydroxylase (TH, Sigma-Aldrich^®^, São Paulo, Brazil) and rabbit anti-glial fibrillar acid protein (GFAP, Sigma-Aldrich^®^, São Paulo, Brazil).

### 2.3. Animals and Ethical Statements

Male Swiss albino mice (25–30 g) were maintained in a temperature and light-cycle-controlled environment with free access to water and food. All procedures were in accordance with the standards of the Brazilian College of Animal Experimentation, and the experimental protocol was approved by the Ethical Committee for Animal Use of the Tiradentes University, Aracaju-SE, Brazil (approval number 010514R).

### 2.4. Samples

The experimental groups were: saline/saline; 6-OHDA/saline; 6-OHDA/BV 0.01 mg·Kg^−1^; 6-OHDA/BV 0.05 mg·Kg^−1^; and 6-OHDA/BV 0.1 mg·Kg^−1^, (*n* = 5) referring, respectively, to the intrastriatal microinjection and the subcutaneous administration. The BV was administered for 3 weeks, once every 2 days (see Section 2.5) [13,14,15].

### 2.5. Induction of Dopaminergic Neuronal Death by 6-OHDA and BV Treatment

The animals were previously anesthetized with ketamine/xylazine (150 and 15 mg·Kg^−1^, respectively, i.p.) and then positioned in the stereotaxic apparatus (Insight^®^, Ribeirão Preto, Brazil). Induction of dopaminergic neuronal death by 6-OHDA was performed by stereotaxic surgery. Briefly, the 6-OHDA (6 μg·μL^−1^ dissolved in 0.9% saline containing 0.02 mg·mL^−1^ of ascorbic acid; Sigma Aldrich^®^) was injected into the right striatum with a 10 μL Hamilton syringe [16,17]. Two injections of 1.5 μL were performed at each site. The stereotaxic coordinates were: (i) AP = +1.0 mm and LL = −2.1 mm from Bregma and DV = −2.9 from the surface of the skull and (ii) AP = +0.3 mm, LL = −2.3 mm, DV = −2.9 mm, with the tooth-bar set at −3.0 mm. The microinjections were performed at a rate of 1 μL·min^−1^ with an infusion pump (Insight^®^, Brazil), and the needle was left in place for an additional 180 s to prevent reflux before being slowly retracted. The movement of an air bubble inside the PE-10 polyethylene tubing connecting the microsyringe with the needle confirmed drug flow. Sham operated (control) animals were submitted to the same procedure but received ascorbate saline (0.02% ascorbic acid) instead of the neurotoxin [16,17]. After the surgical procedure, a suture was performed using a Shalon^®^ (Goiânia, Brazil) suture, nylon 3 cm, cuticular, and an intramuscular injection of a veterinary pentabiotic for small animals (Pencivet Plus PPU, 0.04 mL) was performed. The animals were then maintained in a thermal chamber at 37 °C until recovery from anesthesia.

For the BV treatment, each animal group received a BV dose (0.01 mg·Kg^−1^; 0.05 mg·Kg^−1^ and 0.1 mg·Kg^−1^), respectively, by subcutaneous administration, once every 2 days over a period of 3 weeks [13,14,15].

### 2.6. Behavioral Test for Assessment of Motor Activity: The Open Field Test

The open-field test was used to assess spontaneous locomotor activity, 28 days after lesion. The following parameters were evaluated during 5 min: locomotion or crossings (number of line crosses) and rearings (the number of times the mouse stood on its hind legs) [18]. The open field was made of white colored wood, and it consisted of a quadrilateral with an area of 4.830.25 cm^2^ and walls that were 34.5 cm high, with the base subdivided into sixteen quadrants, visibly marked by lines [19,20].

### 2.7. Histological Analysis

Animals were euthanized in a CO_2_ chamber and their brains were extracted, fixed in formalin and paraffin-embedded. Five micrometer serial sections were cut within a microtome (Leica, São Paulo, Brazil). Neuroanatomical sites (dorsal striatum—CPu; substantia nigra pars compacta—SNc) were identified using a rat brain atlas [21]. The brain sections were stained for tyrosine-hydroxylase (TH), a marker for dopaminergic neurons, and for glial fibrillar acid protein (GFAP), for the evaluation of astrocytes expression. Hematoxylin and eosin (HE) staining was carried out for the histopathological analysis.

### 2.8. Immunohistochemistry for TH and for GFAP

For immunohistochemistry, the antigen recovery was carried out in citrate buffer (pH 6) using a microwave (3 cycles of 5 min). The endogenous peroxidase was blocked (0.3% H_2_O_2_), and tissue sections were incubated over night with the primary antibody (rabbit anti-TH: 1:500, Sigma-Aldrich^®^, São Paulo, Brazil; rabbit anti-GFAP 1:50, Sigma-Aldrich^®^, São Paulo, Brazil). Sections were processed by the avidin-biotin peroxidase system (1:125, Kit ABC, Vectastain, Vector Laboratories^®^ (São Paulo, Brazil), and immune-positive cells were visualized by addition of the chromogen 3,3-diaminobenzidine (DAB, 1 mg·mL^−1^, Sigma-Aldrich^®^, São Paulo, Brazil) and hydrogen peroxide (0.2%). The tissue was washed in phosphate-buffered saline between procedures. Immunopositive cells were revealed by a brown reaction product. The sections were dehydrated in ethanol, cleared in xylene and cover-slipped for optic microscopic observations. In all experiments, tissues from every group were always processed in the same assay [22,23].

### 2.9. Imaging Analysis

The images were captured from the slides using a video camera Olympus C-7070, coupled to microscope Olympus CX31 (Olympus^®^, Tokyo, Japan). The displayed field was recorded and saved. The image was transferred to a microcomputer, adjusted by the editing system and then analyzed by the software “Image J” of National Institute of Health Image (W. Rasband, National Institute of Mental Health). The slices selected for the analysis of SNc, for each animal, corresponded to 2–3 sections adjacent to the plane levels −4.8, −5.4 and –6.1 mm in relation to Bregma, according to the Atlas of Paxinos and Watson (2013) [21]. Every TH-positive cell body comprised into SNc was counted at a 100× magnification. For the quantification of TH-positive fibers in the CPu, a computerized image analysis system was also used, as described above, capable of reading the optical density (OD) in gray levels. For each animal, OD was measured in 2–3 sections adjacent to the plane level 1.3, 0.2 and –1.3 mm in relation to Bregma (Paxinos and Watson, 2013). These distances were assigned arbitrarily, since they represent the rostral, medial and caudal levels, respectively, of this structure. For each level, non-specific labeling was measured by subtracting the OD reading from the corpus callosum. The role dorso-lateral CPu was registered for DO analysis in an area equivalent to 100.000 µm^2^. The quantifications on each side of the striatum and SNc were expressed as a percentage of the contralateral side in the same animals. The GFAP expression in the CPu was expressed by a semi-quantitative score, where 0 was considered as the absence of GFAP positive cells, 1 was considered when 10% or less of the analyzed area was infiltrate, 2 when the infiltrate was seen in 10 to 50% and 3 when GFAP expression was found in more than 50% of the area.

### 2.10. Statistical Analysis

One-way analysis of variance (ANOVA) followed by Tukey’s post-test was performed. Data were expressed as mean ± SEM, and values of *p* ≤ 0.05 were considered statistically significant. All statistical analyses were performed using GraphPad Prism 5.0 software.

## 3. Results and Discussion

### 3.1. Chemical Analysis of Africanized BV (Apis mellífera L.) by HPLC-DAD

**Figure 1:**
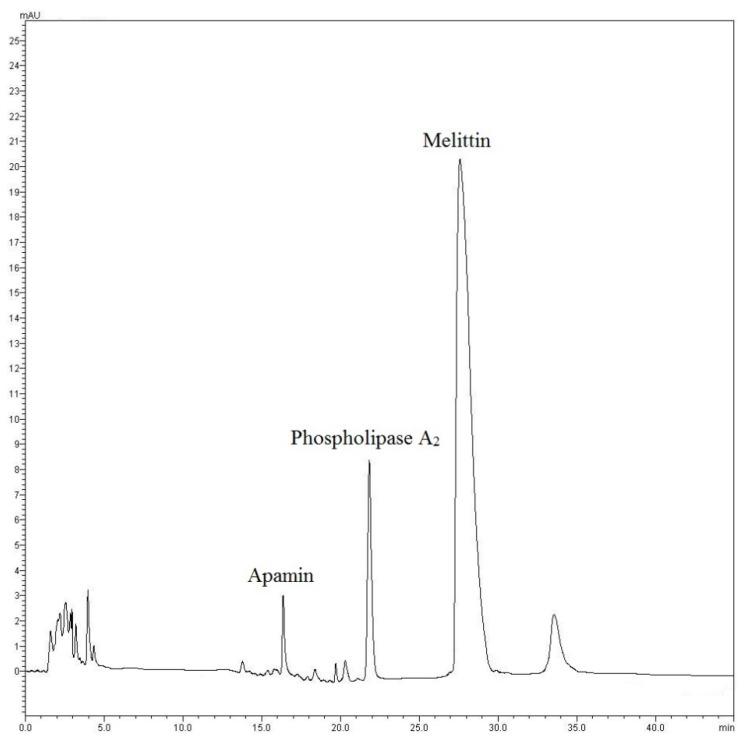
HPLC chromatogram (λ = 280 nm) of the Africanized Bee Venom (*Apis mellífera* L.).

For the determination of melittin in BV, other authors have also used a C18 HPLC column, such as the study recently published by Tanuwidjaja et al. (2021) [24]. In our study, the analysis was carried out following the methods described by Zhou et al. (2010) [25], Dong et al. (2015) [26] and Pereira et al. (2016) [12], with modifications (Section 2.1). The chromatographic analysis of Africanized bee venom revealed the presence of apamin (6.78 ± 0.06 µg·mL^−1^), phospholipase A_2_ (30.63 ± 0.15 µg·mL^−1^) and melittin (137.47 ± 0.13 µg·mL^−1^) (Figure 1), which are quantitatively different from those identified in the venom from European bees [7,14]. To date, there are no studies reporting the use of venom from Africanized bees for the treatment of PD, the second most frequent neurodegenerative disorder in the world [27,28]. Sobral et al. (2016) [29] analyzed samples from the Africanized BV (*Apis mellífera* L.) coming from the northeastern region of Portugal, identifying melittin as the most abundant compound (86.72 ± 0.50 μg.mL^−1^), followed by fosfolipase A_2_ (11.36 ± 0.18 μg.mL^−1^) and apamin (1.80 ± 0.03 μg.mL^−1^), corroborating our results and strengthening the interest of these biomarkers as important pharmacological tools. 

There are two indicators in the manuscript regarding the effectiveness and intensity of dopaminergic lesion (6-OHDA-induced degeneration of the nigrostriatal pathway, Parkinson’s disease model). Motor behavior impairment, assessed in the open field test, is observed after striatal lesion in mice [16,17]. In addition, the loss of dopaminergic cells and fibers was assessed by tyrosine-hydroxylase (TH) immunohistochemistry, a classic marker for dopaminergic neurons. Importantly, animals of the group 6-OHDA treated with vehicle (not BV) presented a significant decrease in crossing, rearing (open field) and TH+ compared to non-lesioned animals.

The animal models available for the study of PD aim to damage the nigrostriatal pathway with toxins or genetic interventions that lead to the loss of dopaminergic neurons and induce motor and non-motor deficits. The 6-OHDA is a catecholamine-selective neurotoxin that reaches dopaminergic neurons via dopamine reuptake transporters, leading to mitochondrial respiratory dysfunction and then to oxidative stress-induced toxicity and neuroinflammation, which causes cell death. It is known that the model induced by 6-OHDA does not reproduce all the characteristics of PD, such as the formation of Levy bodies, but despite this limitation, the relative selectivity of 6-OHDA makes it an interesting alternative to model the classic symptoms of PD. Furthermore, compared to other toxins such as 1-methyl-4-phenyl-1,2,3,6-tetrahydropyridine (MPTP), the 6-OHDA produces robust and relatively stable lesions, with no spontaneous recovery and no risk of contamination for researchers (as is the case with MPTP and pesticides). Significant differences in 6-OHDA doses, injected volumes, and injection sites/coordinates are found throughout the literature, which impact dopaminergic cell survival and behavioral outcomes. When injected into the medial forebrain bundle (MFB) or the substantia nigra pars compacta (SNpc), 6-OHDA induces a massive and rapid lesion in the nigrostriatal pathway, with neurons starting to degenerate within 24 hours. On the other hand, if delivered to the striatum, 6-OHDA induces a slow retrograde degeneration and, consequently, a more progressive depletion of dopaminergic neurons. Therefore, this route of administration offers some advantages over other regions, since a progressive lesion is more relevant in the context of PD. Furthermore, the unilateral administration of 6-OHDA can prevent the high mortality rate observed in bilateral lesions, especially in mice, allowing the use of dosages capable of inducing the motor phenotype, without compromising the welfare of the animals. Studies using genetic models have gained strength in recent years, but it should be noted that the experimental model chosen must be focused on the mechanism that is intended to be addressed, the purpose of the study—in this case, neuroinflammation that results from oxidative stress—and not on mutations that are causally linked to familial cases of PD in humans. Therefore, the 6-OHDA model can mimic not only the behavioral deficits associated with PD but also other histological changes other than nigrostriatal degeneration, such as the glial response [30,31]. 

### 3.2. Assessment of Motor Activity

**Figure 2:**
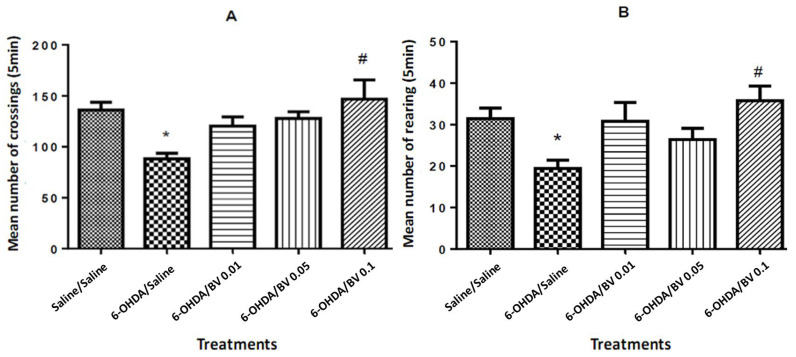
The open field test: effects of the bee venom—BV—(0.01; 0.05 and 0.1 mg·Kg^−1^) on crossing (**A**) and rearing (**B**) behaviors. Bars represent the mean (± SEM) of behaviors expressed in 5 min. * indicates significant difference from saline/saline (*p* < 0.05), and # (*p* = 0.001) indicates significant difference from 6-OHDA/ saline. One-way ANOVA followed by Tukey’s post test.

In the open field test, for the crossings operating motor parameter, it is observed that the group 6-OHDA/saline showed a significant reduction (*p* < 0.05; ANOVA) in relation to the group saline/saline. In contrast, the group 6-OHDA/ BV 0.1 mg·Kg^−1^ significantly increased the crossing compared to the 6-OHDA/ saline (*F*_4–42_ = 4.311; *p* = 0.001; ANOVA) (Figure 2A).

Concerning the rearing behavior, that only the group 6-OHDA/saline presented a significant decrease in relation to the control group (saline/ saline; *p* = 0.03) and the group 6-OHDA/ BV 0.1 mg·Kg^−1^ showed a significant increase in relation to the 6-OHDA/saline group (*F*_4–59_ = 4.192; *p* = 0.001; Figure 2B). 

Regarding the behavioral changes in the open field, mice injured by 6-OHDA and receiving saline (s.c.) showed a decreased mean of crossings and rearing in relation to the saline/saline group. These data are consistent with previous studies [16,17] that used this model of striatal lesion in mice and obtained reduction in motor activity, thus indicating the effectiveness of the lesion produced. This 6-OHDA-induced effect was reversed by the treatment with Africanized BV (*Apis mellífera* L.) at 0.1 mg·Kg^−1^, and an improvement in motor patterns was recorded. This is in agreement with the study by Maurice et al. (2015) [7] that the European BV reverses the action of 6-OHDA on the direct and indirect pathways of the nigrostriatal circuitry. In addition, a previous study performed by Yang et al. (2010) [14] showed that 0.1 μg·g^−1^ of European BV generated increased motor activity and reduced neuroinflammation in animal models of amyotrophic lateral sclerosis (ALS). Although the clinical, neuropathological and debilitating aspects of the PD and ALS are distinct, they share a pattern of neurodegeneration in anatomically or functionally related regions, and there is evidence of a neuroprotective effect of this venom on these diseases [14,32,33].

### 3.3. Histopathological Analysis

**Figure 3:**
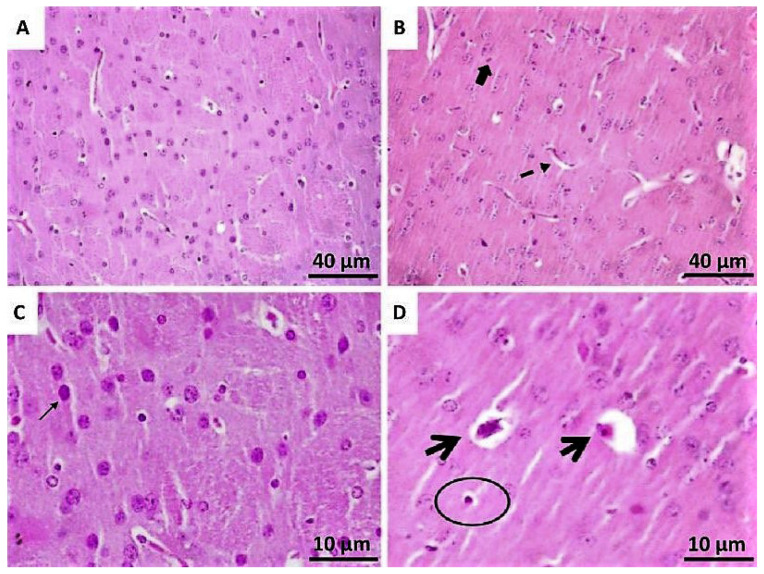
Representative photomicrographs of histological sections stained with Hematoxylin-eosin (HE) showing brain tissue in the striatum—CPu. (**A**,**C**) represent the intact side (controls) with intense cellularity, mainly neuronal (fine arrow), and the images (**B**,**D**) represent the lesioned side. In (**B**), there was an increase in both vascularization (dashed arrow) and presence of microglia (arrow completed). In (**D**), it was observed pyknosis (continuous arrows) and intense nuclear pyknoses accompanied by cytoplasmic retraction, with consequent pericellular halo formation (circle). Magnification of 100× (**A**,**C**) and 400× (**C**,**D**).

The histological analyses carried out in the region of the CPu aimed to evaluate the cellular consequences of the inflammatory process after lesion (Figure 3). In the 6-OHDA/saline group, reduction in cellularity (oligodendrocytes, mainly), increase in the number of blood capillaries and congestion of these capillaries, with nuclei of neurons exhibiting generalized loss of tinctorial affinity, were identified, suggesting possible chromatolysis and assuming granular appearance. 

In addition, cells exhibiting increased cytoplasmic acidophilia, contraction of chromatin, increased density and progressive dissolution of chromatin, with dye affinity loss were identified in the same tested group. There was also intense nuclear pyknoses accompanied by cytoplasmic retraction, with consequent formation of pericellular halo and the intense presence of fibrous astrocytes.

**Figure 4:**
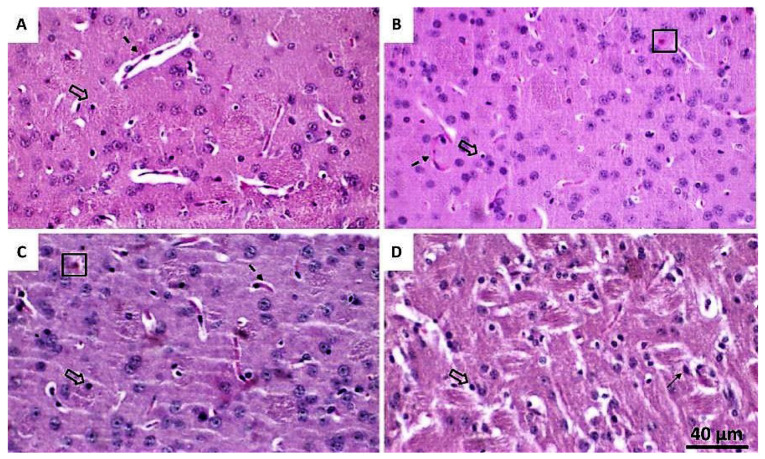
Representative photomicrographs of histological sections stained with Hematoxylin-eosin (HE) showing brain tissue in the striatum—CPu. (**A**) represents the group 6-OHDA/saline; (**B**) represents the group 6-OHDA/ BV 0.01 mg·Kg^−1^; (**C**) represents the group 6-OHDA/ BV 0.05 mg·Kg^−1^; (**D**) represents the group 6-OHDA/BV 0.1 mg·Kg^−1^. Dashed arrows point to blood vessels, thick arrows point to oligodendrocytes, squares highlight necrotic neurons and fine arrows point to healthy neuron. Magnification of 400×.

In the 6-OHDA/BV 0.01 mg·Kg^−1^-treated group, an increase in cellularity (astrocytes, mainly) and reduction in vascularization were observed, along with a remaining presence of necrotic cells and fibrous astrocytes (Figure 4). The presence of necrotic neurons in some areas was also visualized in the 6-OHDA/BV 0.05 mg·Kg^−1^-treated group, which also presented increased vascularization. The group treated with 6-OHDA/BV 0.1 mg·Kg^−1^ presented increased cellularity and minor vascularization.

**Figure 5:**
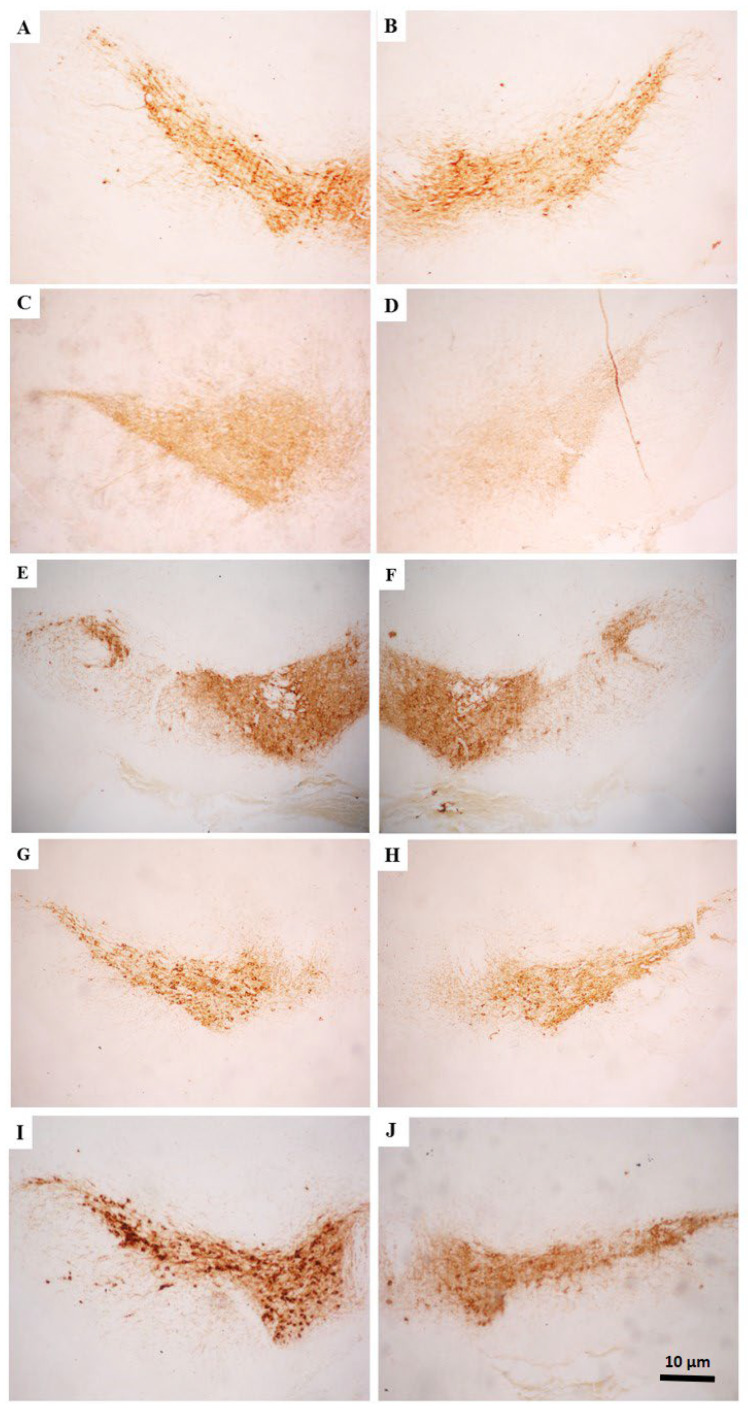
Representative photomicrographs of brain tissue in the substantia nigra pars compacta—SNc—region after immunohistochemistry for TH. The images (**B**,**D**,**F**,**H**,**J**) represent the injured sides, and the images (**A**,**C**,**E**,**G**,**I**) represent their respective controls. In (**A**,**B**): group saline/saline, (**C**,**D**) 6-OHDA/saline, (**E**,**F**) 6-OHDA/BV 0.01 mg·Kg^−1^, (**G**,**H**) 6-OHDA/BV 0.05 mg·Kg^−1^, (**I**,**J**) 6-OHDA/BV 0.1 mg·Kg^−1^, referring respectively to microinjection intrastriatal and systemic treatment. Magnification of 100×.

**Figure 6:**
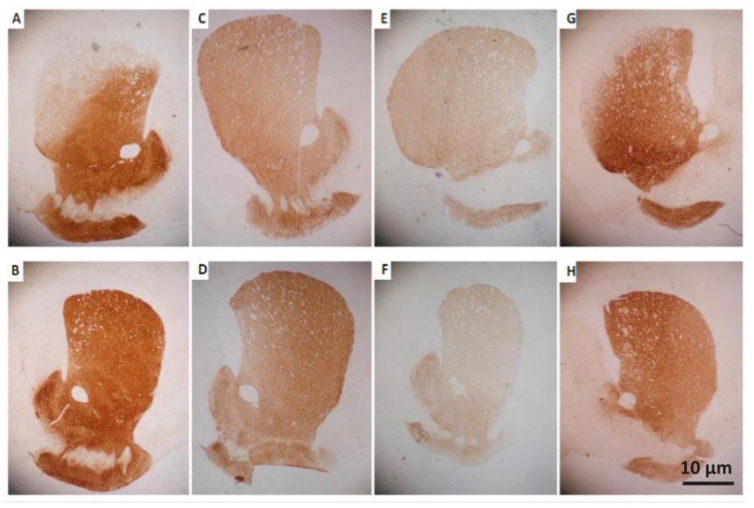
Representative photomicrographs of brain tissue in the dorsal striatum—CPu—after immunohistochemistry for TH. The images (**A**,**C**,**E**,**G**) represent the injured sides, and the images (**B**,**D**,**F**,**H**) represent their respective controls. In (**A**,**B**): group 6-OHDA/saline, (**C**,**D**): 6-OHDA/BV 0.01 mg·Kg^−1^, (**E**,**F**): 6-OHDA/BV 0.05 mg·Kg^−1^ and (**G**,**H**): 6-OHDA/BV 0.1 mg·Kg^−1^, referring respectively to microinjection intrastriatal and systemic treatment. Magnification of 40×.

The qualitative analysis of TH expression showed that the animals from the 6-OHDA/saline group presented a reduction in the number of positive neurons in the SNc, when comparing the ipsilateral (lesioned) side to the contralateral (control). In groups treated with BV, this effect was not that evident. Instead, both sides were found to be quite similar (Figure 5). The same was noticed regarding the TH-positive fiber density in the CPu (Figure 6).

**Figure 7:**
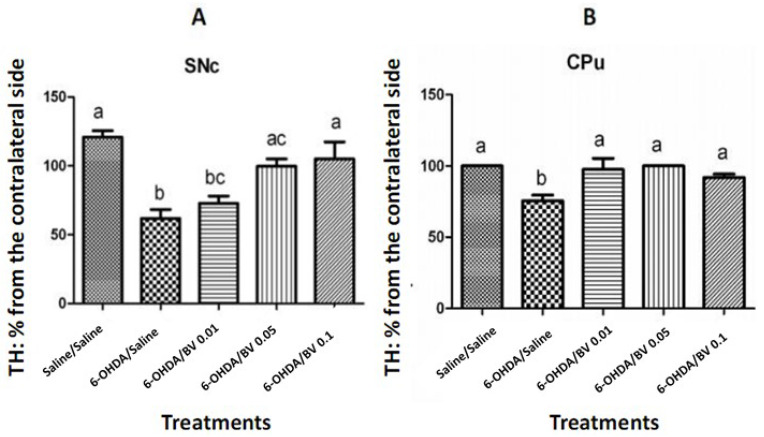
Percentages of tyrosine-hydroxylase-positive cell density (TH^+^) in the: (**A**) substantia nigra pars compacta (SNc) and (**B**) striatum (CPu). Different letters indicate statistical difference between groups in SNc (*p* < 0.0001) and in Cpu (*p* = 0.001), while equal letters indicate similar results. BV: bee venom; 6-OH: 6-hydroxydopamine. Columns represent the mean and bars represent the S.E.M. One-way ANOVA followed by Tukey’s post test.

The quantification of TH-positive cells in the SNc highlighted a significant reduction in the mean percentage of the remaining dopaminergic neurons in the 6-OHDA/saline group (*p* < 0.0001). In contrast, the lesioned animals that received BV from 0.05 to 0.1 mg·Kg^−1^ showed a significant increase in the mean percentage of remaining cells, compared to those treated with 6-OHDA/saline (*p* < 0.0001, Figure 7A, one way ANOVA, *F*_4–70_ = 7.794, *p* < 0.0001).

The animals treated with 6-OHDA/saline showed loss of dopaminergic terminals in the CPu, as verified by a significant decrease in the mean percentage of immunoreactivity for TH in this region compared to the saline/saline group (*p* = 0.001). On the other hand, animals that received BV in all tested doses showed a significant increase in the mean values of optical density (OD) compared to the 6-OHDA/saline treatment (*p* = 0.001, Figure 7B, one way ANOVA, *F*_4–33_ = 5.719; *p* = 0.001).

In addition, we found that Africanized BV reversed the 6-OHDA-induced loss of TH-positive neurons in the SNc (0.05–0.1 mg·kg^−1^) and loss of dopaminergic terminals in the CPu (from 0.01 to 0.1 mg·kg^−1^). 

The analysis of TH expression showed that a moderate lesion was induced, compatible with the initial stages of PD [34], since 6-OHDA caused a reduction in the number of TH immunoreactive cell bodies about or higher than 50%. In the lesion model used here, 6-OHDA was injected into the dopaminergic terminals of the striatum, unilaterally, which causes a less extensive and progressive reaction that was more similar to the development of PD [35]. The subsequent retrograde cell death evolves from one to three weeks after the lesion [2]. 

Cheng et al. (2010) [36] performed quantitative morphological studies that revealed a remarkable consistency in the relation between neuronal loss in the SNc and the appearing of motor symptoms. Tagliaferro and Burke (2016) [34] reported that about 30% of dopaminergic neurons SNc and up to 50% of the striatum dopaminergic terminals are lost when the early symptoms appear in PD. Then, there is a close relationship between behavioral changes and the extent of dopaminergic denervation, and it is possible that the reductions seen in the open field test reflect the loss of dopaminergic cells.

Since BV is neuroprotective of both neuronal and behavioral performance, the association between behavioral responses and the actions of BV compounds should be disclosed. Melittin, the major compound of BV, shows important effects by inhibiting neuroinflammatory mechanisms [32,37,38]. 

In pathological conditions (e.g., lesions in brain tissue), microglia are rapidly activated and produce inflammatory mediators such as tumor necrosis factor α (TNF-α), interleukin-6 (IL-6) and IL-1β. The expression of these pro-inflammatory cytokines is regulated by the nuclear transcription factor-kappa B (NF-κB), which accelerates the neurodegenerative processes [39].

Melittin suppresses the expression of nitric oxide (NO) and inducible nitric oxide synthase (iNOS), blocking lipopolysaccharide (LPS)-induced activation and the induction of inflammatory cytokines by NF-κB in microglial cells. Melittin suppresses the expression of COX-2 and prostaglandins, resulting in anti-inflammatory properties [35]. In addition, BV increased the levels of the anti-inflammatory IL-10, which has receptors on neurons and microglia and reduced IL-6 levels after traumatic spinal cord injury with potent anti-inflammatory activity [40].

Calcium-activated potassium channels (SK) are also potential targets in the treatment of PD, since they produce the post-potency hyperpolarization phase, which contributes to the control of the action potential frequency and neuronal firing and are also involved in synaptic plasticity [41,42]. The SKs have three subunits widely expressed in limbic structures and basal ganglia [7,42]. In particular, SK3 controls the stability of the endogenous activity of dopaminergic neurons in the SNc and in the ventral tegmental area (VTA). Apamin, a polypeptide that corresponds to 2–3% of the dry weight of the venom, prevents depolarization and blocks SK in the membrane, preventing the physiological action of adrenaline from opening this channel [6,25,43]. 

Mourre et al. (2017) [44] evaluated whether SK channel expression in the basal ganglia is modified in Parkinsonian rats and how this may affect behavioral performance in a reaction time task. The authors noticed a reduction in the level of expression of the SK3 channel in the SNc, with improvement in motor activity, after apamin channel block.

In contrast, Chen et al. (2014) [6] identified that apamin, a polypeptide equivalent to 2–3% of the dry weight of the venom, reversed the motor deficiency induced by unilateral dopaminergic lesions by 6-OHDA concentrations of 0.1 and 0.3 mg·Kg^−1^ and increased dopamine concentrations in the striatum after injury, so that administration of this compound alone was satisfactory for restoration of motor function in this animal model.

The BV blocks the calcium-activated potassium channels, thus restoring the functional balance in the core sub-circuits of the nucleus basalis [7]. Thus, pharmacological blockade of these channels is capable of reversing Parkinsonian motor deficits and promoting dopaminergic neuronal activity [6].

In the context of neuroinflammation, it is pertinent to discuss the cellular consequences resulting from the inflammatory mechanism to which the tissue was exposed. The results regarding CPu analysis of the lesion and control groups corroborate other findings [45,46,47].

After 28 days of striatum lesion by 6-OHDA, Silva-Adaya et al. (2014) [45] identified extensive damage in the lesion group, followed by abundant pycnotic nucleus, which refers to condensed chromatin due to a pathological process. Marei et al. (2015) [46] reported massive neuronal cytotoxicity with neuronal degeneration, striatum shrinkage, volume reduction and enlargement of the lateral ventricles 8 weeks after injury.

Because it is a progressive lesion, it is estimated that the longer the duration the greater the damage to brain tissue, suggesting intense neurodegeneration. In the study of Nagappan and Krishnamurthy (2016) [47], intense cytoplasmic vacuolization was observed in the region of the CPu 45 days after injury. In the study by Marei et al. (2015) [46], medium and large neurons with hyperchromatic nuclei and size reduction were identified. Multinucleated giant cells, vacuolization and blood capillary congestion were also visualized.

The histological analysis of the lesion group also presented an intense reduction in the number of cells in general and an apparent increase in the lateral ventricles, chromatolysis and necrosis. In contrast, the BV in a dose of 0.1 mg·Kg^−1^ generated an increase in cellularity, with similar appearance to the control group, and lower vascularization, suggesting preservation of cellular and tissue characteristics. 

The lower tested dose generated a glial reaction in the ipsilateral striatum, with a strong presence of necrotic cells, denoting that the venom did not prevent neuronal death but caused intense astrocytic reaction aiming at the maintenance of neuronal homeostasis. Considering that astrocytes are also vulnerable to oxidative stress (which results from the action of 6-OHDA, due to the low levels of the enzymes superoxide dismutase, catalase and oxidized glutathione, which make them susceptible to damage [48]), the maintenance of these cells plays an important role in the preservation of synaptic plasticity and as a defensive mechanism for cells exposed to oxidation and inflammation [49]. Thus, the BV acts as an anti-inflammatory agent, blocking these events, starting from the lowest tested dose, still in a non-effective way, but with tissue preservation at the highest evaluated dose (0.1 mg·Kg^−1^).

**Figure 8:**
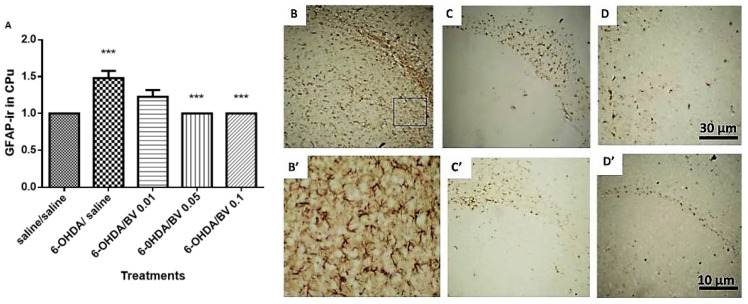
Quantification of GFAP-ir density in the CPu ipsilateral to the lesion (**A**). Representative photomicrographs of brain tissue in the dorsal striatum—CPu—region after immunohistochemistry to GFAP. In (**B**): 6-OHDA/saline group, (**B’**): 6-OHDA/saline with magnification of 400×, (**C**): saline/saline, (**D**): 6-OHDA/BV 0.01 mg·Kg^−1^, (**C’**): 6-OHDA/BV 0.05 mg·Kg^−1^ and (**D’**): 6-OHDA/BV 0.1 mg·Kg^−1^, referring respectively to intrastriatal microinjection and systemic treatment. Magnification of 40× (***, *p* < 0.001).

The qualitative analysis of GFAP expression indicates that the brains of animals receiving 6-OHDA/saline presented astrocytic infiltrate in the CPu. It was not observed in the groups treated with BV, which were similar to the control (Figure 8). The quantification of GFAP in the CPu showed a significant increase in the mean of astrocytic infiltrate in the 6-OHDA/saline group (*p* < 0.001). In contrast, the lesioned animals that received BV from 0.05 to 0.1 mg·Kg^−1^ showed a significant decrease in the mean of GFAP, compared to those treated with 6-OHDA/saline (*p* < 0.001, Figure 8).

With regard to the expression of GFAP, the immunohistochemistry qualitative analysis corroborates the histochemical findings, indicating presence of reactive astrocytosis in the lesion group, indicative of inflammation of brain tissue and abnormal changes in surrounding cells. The same findings were observed by Tripanichkul and Jaroensuppaperch (2013) [50], which identified increased immunoreactivity by GFAP in the striatum ipsilateral to the lesion. 

Injection of 6-OHDA into the striatal tissue aims to degenerate the synaptic terminals of the dopaminergic neurons. Through this lesion, the astrocytes become activated and acquire hypertrophic morphology, increasing the production of its intermediate filament, GFAP [51]. 

This procedure is followed by microglial activation due to non-specific tissue damage at the site of injection and the cytotoxic effects of the drug [52]. Some factors (e.g., needle penetration, high hydrostatic pressure generated by the injection or high concentration of 6-OHDA around the bevel of the needle) initiate microglial activation, which is intensified over time [53].

Upon administration of BV, reduced immunoreactivity is observed, with similar feature to the control group at 0.1 mg·Kg^−1^. This finding raises discussion about the action of apamin, which blocks the calcium-activated potassium channels and around of astrocytes [54]. SK3 contributes to the coordination of membrane excitability and to the regulation of the resting and control potential of spontaneous impulse generation.

Thus, once absorbed and metabolized by the astrocytes, there is a change in the concentration of extracellular ions and consequent repercussion on the potential of the membrane [55]. Thus, it is suggested that a lower dose of BV influences SK3, reducing astrocytic marking.

PD is a multifactorial disease involving aging, genetics and environmental factors, and therapies directed to protein aggregation/gene mutations are one of many aspects that deserves attention. Mitochondrial dysfunction, oxidative stress, apoptosis, imbalance in calcium homeostasis and neuroinflammation are also hallmarks of PD. To date, the dopaminergic replacement by levodopa orally is still the gold standard treatment for PD. However, it is only a symptomatic therapy and induces dyskinesia as a side effect [56]. To find new multi-target drugs or therapies (preferably without adverse reactions) is a great challenge for PD and it is noteworthy that BV is a complex mixture of neuroprotective elements, which could be interesting to target multiple aspects of the cell death cascade events. In fact, studies in humans and clinical trials have shown that BV may be beneficial in the treatment of disorders related to microglial activation, such as PD [57,58]. 

## 4. Conclusions

The Africanized BV (*Apis mellifera* L.) presented large amounts of melittin, phospholipase A_2_ and apamin. The BV reversed the 6-OHDA-induced decrease in spontaneous motor activity (0.1 mg·kg^−1^), loss of TH positive neurons in the SNc (0.05–0.1 mg·kg^−1^) and dopaminergic terminals in CPu (from 0.01 to 0.1 mg·kg^−1^) and astroglial reaction (0.05–0.1 mg·kg^−1^). We found that BV promoted motor recovery (behavioral test), protection of dopaminergic neurons from 6-OHDA in the nigrostriatal pathway and prevented astroglial reaction (immunohistochemical analysis of TH+ and GFAP+ cells, respectively), anticipating our assumption that the peptide-rich Africanized BV presents neuroprotective effects in a progressive mouse model of PD. Future in vivo and in vitro studies are panned to better understand the cellular and molecular mechanisms of the neuroprotection by BV (which inflammatory and oxidative pathways, enzymes, genes, proteins, etc.). It would be interesting to investigate the actions of the isolated melittin on the 6-OHDA lesion model (to date, in vivo studies were conducted on MPTP-induced PD [59]). Moreover, although bee venom therapy (acupuncture mainly) has been studied in human PD and other diseases with promising outcomes, it can cause adverse effects, even rarely, including anaphylaxis [60]. Thus, studies using nanotechnology (controlled drug delivery systems, increased bioavailability and target selectivity, lower dosage) targeting the delivery of BV to the nervous system would bring a great improvement in this field. Finally, considering the etiology underlying the dyskinesias (side-effect of the gold standard treatment for PD), i.e., oxidative stress, and the biological activities described for BV, it opens a window to explore its antidyskinetic effect.

## Data Availability

Data are available from corresponding authors upon request.
